# Validating the satisfaction with life scale among early adolescents: psychometric assessment using item response theory

**DOI:** 10.1007/s12144-025-07518-3

**Published:** 2025-03-01

**Authors:** Scott I. Donaldson, Trista A. Beard, Daniel Soto, Ryan Lee, Adam M. Leventhal, Jennifer B. Unger

**Affiliations:** 1 Department of Population and Public Health Sciences, Keck School of Medicine, University of Southern California, Los Angeles, CA USA; 2 1845 N Soto Street, 3rd Floor, SSB 310-23, Los Angeles, CA 90032 USA

**Keywords:** Subjective well-being, Satisfaction with Life Scale, Item response theory, Adolescents, Substance use

## Abstract

The Satisfaction with Life Scale (SWLS) has been extensively validated using Classical Test Theory, mostly relying on factor analytic methods among adult samples. The current study used Item Response Theory to validate SWLS among a sample of early adolescents in California and examine associations between SWLS and tobacco and cannabis use. Data were collected from ninth-grade students (*N* = 2552) attending 10 public high schools in Los Angeles, California in 2013. Nonparametric and parametric item response modeling were used to validate the SWLS. Binary logistic regression was used to examine the associations between SWLS and tobacco and cannabis use. Item discrimination indices were above 1.80, indicating all items functioned appropriately in terms of measuring the construct and separating participants of different levels of life satisfaction. The test information curve indicated that the SWLS was best for discriminating between respondents with low to average life satisfaction. Participants who reported high scores on the SWLS, compared with those who reported low scores, were significantly less likely to report lifetime e-cigarette, cigarette, and cannabis use. The SWLS possessed excellent psychometric properties among a sample of early adolescents in California. Findings may be useful for scholars and practitioners to screen for subjective well-being in early adolescence, and target interventions focused on improving adolescent health & well-being, which may help prevent substance use initiation or sustained use.

## Introduction

The science of subjective well-being (SWB) has grown considerably over the past few decades, amassing thousands of peer-reviewed publications each year (Diener et al., 2018). Subjective well-being has been defined as an individual’s global emotional and cognitive evaluation of their life (Das et al., 2020). Several measures have been developed and validated to assess SWB, such as Cantril’s Ladder, the Positive and Negative Affect Schedule (PANAS), and the Scale of Positive and Negative Experience (SPANE), among others (Pavot & Diener, 2008). However, the Satisfaction with Life Scale (SWLS) has emerged as one of the most widely used measures of SWB, specifically focusing on the cognitive component through its assessment of global life satisfaction (Diener et al., 1985). This 5-item measure is notable for its brevity and simplicity, providing a clear and efficient tool for capturing individuals’ evaluative judgments about their lives. Its adaptability and strong psychometric properties make it particularly valuable across diverse populations and research contexts.

The psychometric properties of the SWLS have been extensively validated among adult samples, showing adequate internal consistency, measurement invariance, and discriminant and predictive validity with outcomes, such as health and longevity, social relationships, and work performance (Diener et al., 2018). The psychometric properties of the SWLS have also been examined among adolescent samples and have been associated with school performance (Shek & Li, 2016), physical activity (Piko & Keresztes, 2006), acculturation (Virta et al., 2004), and emotional intelligence (Rey et al., 2011). These associations are important, not least because adolescence is a period of heightened vulnerability where many of the factors that contribute to well-being throughout the lifespan are developed and established (Ross et al., 2020). Subjective well-being may serve as a protective factor for adolescents, increasing their positive psychosocial, educational, and health outcomes, while mitigating multiple health risk behaviors, such as tobacco and cannabis use (Mohamad et al., 2018). For example, research has shown that low life satisfaction was associated with greater odds of reporting tobacco, alcohol, and marijuana use (Lew et al., 2019). However, it is unclear if these associations extend to new and emerging popular products like electronic (e-cigarettes) and modern cannabis, which tend to have much higher potency for addiction than previous years (Jankowski et al., 2019).

While a few studies have used IRT to examine SWLS (Diener et al., 2018), to our knowledge, IRT has not been applied to the measurement of the SWLS among adolescents in California–one of the most populous and diverse states in the country, with nearly 9 million adolescent residents (Statista, 2024). Most validation studies of SWLS have relied on methods like factor analysis to provide psychometric support. While CTT is important for designing and validating new psychological tests, it is limited in its ability to differentiate between individuals who may be high or low on a given latent trait (i.e., the construct of interest). For example, CTT does not provide estimates of how each item on a scale may impact the overall score (Diener et al., 2018). Item Response Theory (IRT), in contrast, can show where a scale is suitable for participants at different levels of the latent trait (i.e., test information for very high or low scorers). Additionally, CTT assumes that errors of measurement are constant across all participants, whereas IRT can show how error varies along the latent trait, and which items (if any) may poorly contribute to the psychometric validity of a scale (Zanon et al., 2016). Item response theory can provide item-level and overall scale-level information, enabling researchers to establish predictive validity with desired outcome measures.

The current study used IRT to examine the psychometric properties of the SWLS, including an assessment of item discrimination and difficulty, conditional reliability, and test information. The current study also provided preliminary associations between the SWLS and lifetime e-cigarette, cigarette, and cannabis use. Findings may be useful for scholars and prevention programmers to screen for well-being in early adolescence and target interventions focused on improving adolescent health and well-being and preventing substance use initiation.

## Methods

### Participants and procedure

Data were collected from Wave 1 (Fall 2013) of an ongoing longitudinal survey on substance use and mental health among students attending ten high schools in Los Angeles County, California (Peters et al., 2018). Around 40 public high schools in the Los Angeles metropolitan area were invited to participate in this study. These schools were selected based on their diverse demographic profiles and convenient geographic location. Details on study design and methods can be found elsewhere (Leventhal et al., 2015). Most participants reported that their gender was female (*n* = 1419, 55.6%) followed by male (*n* = 1113, 44.4%). The most commonly reported race/ethnicity was Hispanic or Latino (*n* = 1186, 46.5%), followed by Asian (*n* = 462, 18.1%), White (*n* = 403, 15.8%), multiracial (*n* = 152, 6.0%), other (*n* = 143, 5.6%), Black or African American (*n* = 118, 4.6%), and Native Hawaiian or Pacific Islander (*n* = 88, 3.4%). Participant sociodemographic characteristics can be viewed in Table 1. Paper-and-pencil surveys were administered in the classroom and the survey took approximately 20 minutes to complete. Participating students provided active written or verbal assent and their parents provided active written or verbal consent. Students who were absent from class on the day of data collection completed the survey via telephone or online. The University of Southern California Institutional Review Board approved the study.

**Table 1:** Participants sociodemographic characteristics (*N* = 2552)

Demographic variable	*n*	%
Gender		
Female	1419	55.6
Male	1133	44.4
Race/ethnicity		
Asian	462	18.1
Black or African American	118	4.6
Hispanic or Latino	1186	46.5
Native Hawaiian or Pacific Islander	88	3.4
White	403	15.8
Multiracial	152	6.0
Other	143	5.6
Home language		
Only English	848	33.2
Mostly English	568	22.3
English and another language	1046	41.0
Mostly another language	14	0.50
Only another language	3	0.10
Clinical depression	73	2.9
No	1639	64.2
Yes	913	35.8

### Measures

#### Satisfaction with life scale

The SWLS was used to measure the global cognitive component of SWB using 5-items on a seven-point Likert-type scale (1 = strongly disagree; 7 = strongly agree) (Diener et al., 1985). Example items included “In most ways my life is close to ideal” and “the conditions of my life are excellent.” Research has supported the psychometric properties of the SWLS among adolescents, including excellent internal consistency, test-retest reliability, predictive and concurrent validity, as well as measurement invariance across gender (Jovanović, 2016). Based on past research (Diener et al., 1985), a total SWLS score was computed out of a possible 35 points. To analyze the strengths of associations with substance use, the SWLS total score was recoded as a binary score. Participants who reported a score less than or equal to 20 (i.e., average of ‘4’ slightly agree on each item) were classified as low, while participants who reported a score greater than 20 were classified as high.

#### Sociodemographic and clinical covariates

Past research has indicated that males and certain racial/ethnic groups, such as Black or African American and Hispanic adolescents, are more likely to engage in substance use (Delahanty et al., 2019). As such, gender was included as a sociodemographic covariate and coded as female or male. Race/ethnicity was coded as Asian, Black or African American, Hispanic or Latino, Native Hawaiian or Pacific Islander, White, Multiracial, or other. Participants were asked to indicate whether they had ever been diagnosed with clinical depression, which was coded as “1”(yes) or “0”(no).

#### Tobacco and cannabis use

Informed by prior research (Eaton et al., 2010), participants were asked to indicate whether they had ever used the following substances in their lifetime: e-cigarettes (“1”(yes) or “0”(no)), cigarettes (“1”(yes) or “0”(no)) or cannabis (“1”(yes) or “0”(no)).

## Analytic strategy

All analyses were conducted in the *R* statistical software using the *mirt*, *psych*, *lordif*, *mokken*, and *stats*packages (Chalmers & Chalmers, 2012; Choi et al., 2011; R Core Team, n.d.; Revelle, 2017; van der Ark, 2007). Descriptive statistics, including means, standard deviations, response frequencies, and skewness and kurtosis were computed. Past research has suggested that data is considered to be normal if skewness is between−2 to + 2 and kurtosis is between ‐7 to + 7 (Byrne, 2010). Based on previous research suggesting that sufficient statistical power and the assumptions of missing data being completely at random are satisfied in this dataset (Kang, 2013), the missing data was handled using listwise deletion (*n* = 588).

Before implementing a parametric IRT model, a Mokken Scale Analysis was used to examine the dimensionality of the SWLS (Mokken, 2011). To assess unidimensionality, item-pair scalability coefficients were computed using coefficient H. A strong unidimensional scale is denoted by H > 0.50, 0.40 < H < 0.50 denotes a medium scale, and H < 0.40 denotes a weak scale (Mokken, 2011). An automated item selection procedure was used to calculate inter-item covariances and the relationship between items and the latent trait (Meijer et al., 2004). Latent monotonicity for each item was examined using a visual plot of the item step response function by rest score group. The rest score group is achieved by summing the overall score minus the score on each item (Junker & Sijtsma, 2000). Using past research (Robinson et al., 2019), a residual correlation matrix with a cutoff < 0.25 was used to assess local independence between the SWLS items.

A Samejima’s graded response model was used to estimate item discrimination and ability on the latent trait of the SWLS (Woods, 2006). The graded response model is an extension of the two-parameter logistic model for items with two or more response categories (Samejima, 1997). Three parameters were estimated and used to evaluate each item of the 5-item measure of the SWLS: 1): the ability level of SWLS was denoted as theta (*Θ*); 2) threshold values were denoted as *b*_*i*_, with items that represent the top end of the SWLS being considered more difficult; 3) the discrimination of the item was denoted as *a.* Items that accurately differentiate an individual who is low on dimensions of the SWLS from individuals who are high on dimensions of the SWLS received a higher discrimination score. Test information was used to estimate the scale’s precision across the latent trait, and option characteristics curves were used to screen for the ability of response options on each item to progress along the latent trait given the response categories (i.e., 1–7).

While dichotomization may reduce sensitivity to individual differences, it simplifies interpretation and facilitates practical applications for identifying individuals at risk in large datasets. As such, binary logistic regression was used to examine the association between the SWLS score and lifetime e-cigarette, cigarette, and cannabis use. Odds ratios and 95% confidence intervals were computed. Based on prior research documenting the role of gender, race/ethnicity, and clinical depression on substance use, such variables were included as covariates (Fonseca et al., 2021; Wu et al., 2011; Quello et al., 2005).

## Results

### Descriptive statistics

Item means, standard deviations, and response frequencies for the 5-item measure of the SWLS are presented in Table 2. Skewness and kurtosis values were considered normal. The mean scores for SWLS ranged from 3.9 to 5.2. The item “I am satisfied with life” had the highest average score (M = 5.2, SD = 1.7), whereas the item “If I could live my life over, I would change almost nothing” had the lowest average score (M = 3.9, SD = 2.0). All items were included for item response modeling.

**Table 2:** 5-item satisfaction with Life Scale with item means, standard deviations, and response frequency values

Dimension	Item	M (SD)	Response frequency						
			1	2	3	4	5	6	7
Ideal	In most ways my life is close to ideal.	4.7 (1.6)	127	173	186	603	585	712	259
Excellent	The conditions of my life are excellent.	5.0 (1.7)	105	165	241	348	535	739	512
Satisfied	I am satisfied with life.	5.2 (1.7)	121	154	199	307	363	783	718
Important	So far I have gotten the important things I want in life.	5.0 (1.7)	133	174	208	336	509	708	577
Change	If I could live my life over, I would change almost nothing.	3.9 (2.0)	442	320	411	286	418	386	382
*M *mean; *SD *standard deviation; *N* = 2552									

### Dimensionality

A Mokken Scale Analysis of the 5-item measure of the SWLS had a strong item-pair scalability coefficient (*H* = 0.614). An automated item selection procedure and visual inspection of plots further supported the dimensionality of the 5-item measure of SWLS, by showing a monotonic relationship with the rest score group. All residual correlations between items were below the recommended < 0.25,^31^ suggesting local independence.

## Item response modeling

Table 3shows the graded response model item parameters for the 5-item measure of SWLS. The discrimination parameters for the five items used to measure SWLS ranged from 1.8 to 4.2, which is considered very high discrimination (Baker, 2001). The difficulty thresholds (i.e., the point at which each item is 50% likely to be endorsed from the preceding response options along the continuum of theta) 1 versus 2 ranged (*b*_1_) from − 2.3 to −2.0, 2 to 3 (*b*_2_) ranged from − 1.6 to −0.76, 3 to 4 (*b*_3_) ranged from − 1.2 to −0.20, 4 to 5 (*b*_4_) ranged from − 0.55 to 0.17, 5 to 6 (*b*_5_) ranged from − 0.15 to 0.76, 6 to 7, and (*b*_6_) ranged from 0.64 to 1.7.

**Table 3:** Graded response model item parameters for the 5-item satisfaction with Life Scale

Item	a	b_1_	b_2_	b_3_	b_4_	b_5_	b_6_
In most ways my life is close to ideal.	2.0	−2.3	−1.6	−1.2	−0.30	0.41	1.7
The conditions of my life are excellent.	3.5	−2.0	−1.4	−0.93	−0.47	0.01	0.94
I am satisfied with life.	4.2	−1.9	−1.4	−0.99	−0.55	−0.15	0.64
So far I have gotten the important things I want in life.	2.4	−2.0	−1.5	−1.0	−0.54	0.06	0.94
If I could live my life over, I would change almost nothing.	1.8	−1.3	−0.76	−0.20	0.17	0.76	1.5
*a* = discrimination; *b* = difficulty; *N* = 2552							

**Fig. 1:**
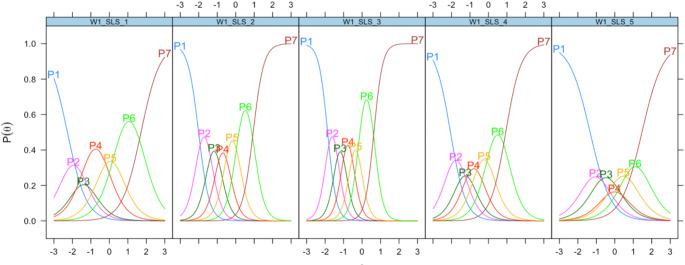
Option characteristic curves for the 5-item measure of the Satisfaction with Life Scale. Note. P1 = the probability of respondents endorsing response option 1 (strongly disagree); P7 = the probability of respondents endorsing the response option 7 (strongly agree); W1_SLS_1 = Ideal; W1_SLS_2 = Excellent; W1_SLS_3 = Satisfied; W1_SLS_4 = Important; W1_SLS_5 = Change

Figure 1 shows the option characteristic curves for the 5-item measure of SWLS.

**Fig. 2:**
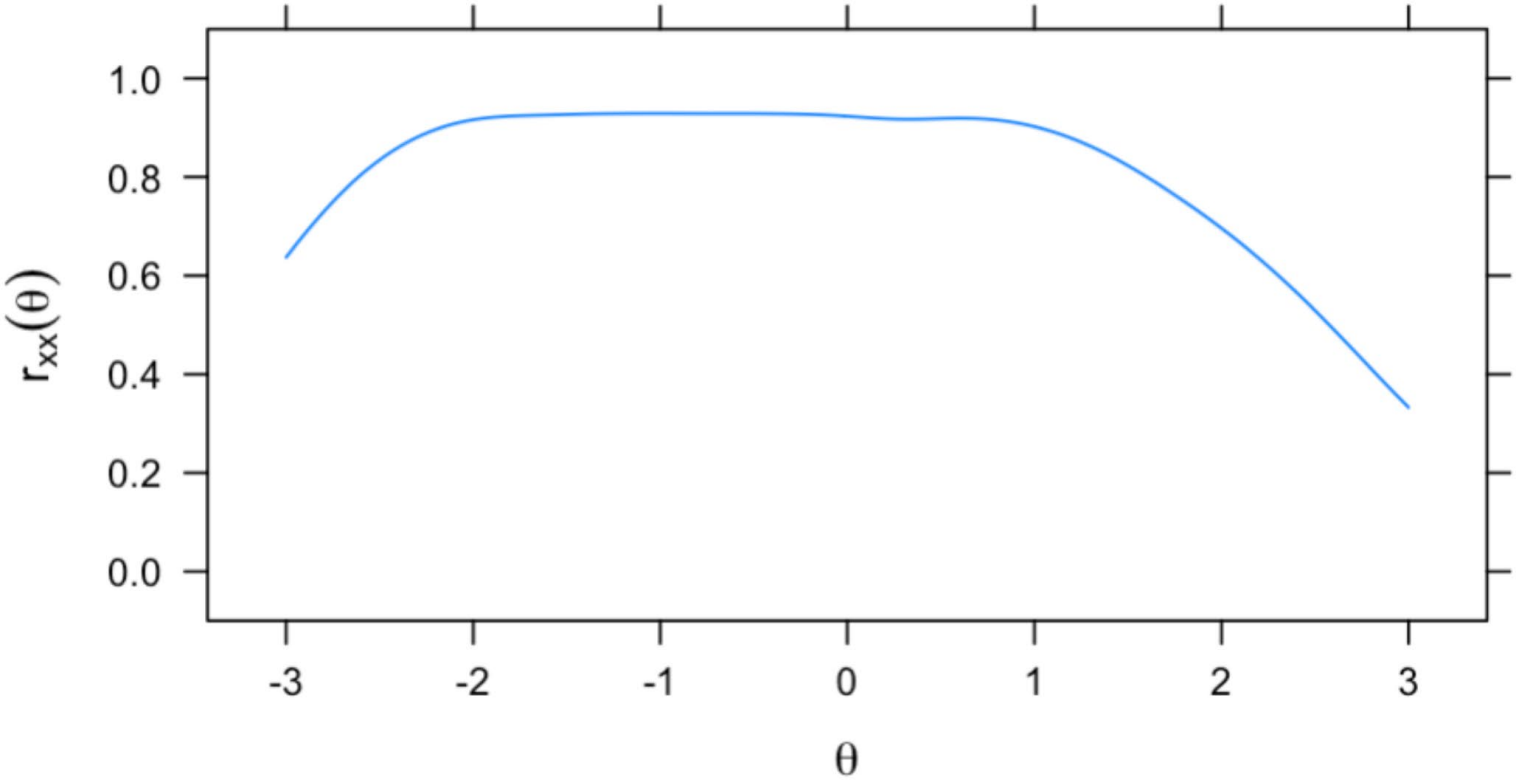
Conditional reliability plot of the 5-item Satisfaction with Life Scale. Note: Θ = the level of life satisfaction; r_*xx*_ = reliability value from 0 to 1

**Fig. 3:**
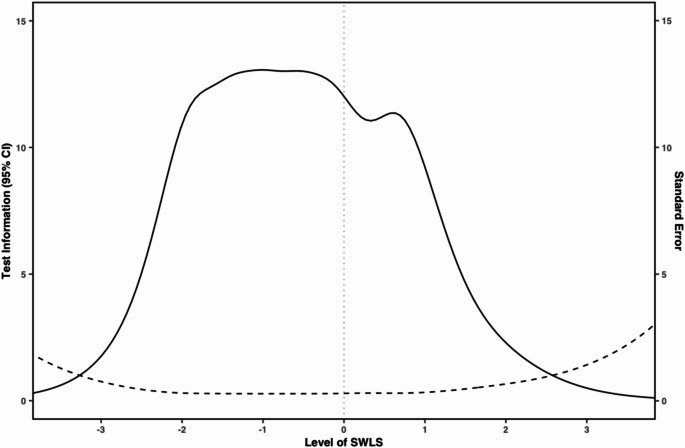
Test information function for the 5-item measure of Satisfaction with Life. Note: Information is an arbitrary value estimated using the latent trait

Items that had higher discrimination (e.g., “I am satisfied with life.” [*a* = 4.2], “The conditions of my life are excellent.” [*a* = 3.5]) were endorsed along theta as the response options increased. On the other hand, items that had lower discrimination (e.g., “If I could live my life over, I would change almost nothing.” [*a* = 1.8]) were less effective at distinguishing between response options as theta increased. Figure 2 shows a conditional reliability plot of the 5-item measure of SWLS. Visual inspection shows that score estimates were most reliable in the − 3 to + 1 theta range. Figure 3 shows the test information function for the 5-item measure of SWLS. Visual inspection shows that most test information was observed in the − 2 to 1 theta range, with a slightly lower amount of test information contained between the average theta level and + 1 standard deviation above the mean.

## Concurrent validity

The associations of the binary recoded SWLS 5-item composite score into high vs. low with e-cigarettes, cigarettes, and cannabis are shown in Table 4. Participants who reported high scores on the SWLS, compared to those who reported low scores, were significantly less likely to report lifetime e-cigarette use (AOR = 0.66, 95% *CI*=[0.51, 0.85]), cigarette use (AOR = 0.63, 95% *CI*=[0.46, 0.88]), and cannabis use (AOR = 0.73, 95% *CI*=[0.55, 0.98]) after adjusting for other sociodemographic and clinical covariates.

**Table 4:** Adjusted associations between the satisfaction with Life Scale and lifetime tobacco and cannabis use

Regressors	E-cigarettes		Cigarettes		Cannabis	
	AOR	95% CI	AOR	95% CI	AOR	95% CI
Satisfaction with Life Scale						
High vs. low	0.66	[0.51, 0.85]	0.63	[0.46, 0.88]	0.73	[0.55, 0.98]
Covariates						
Ethnicity						
Asian vs. White	0.80	[0.54, 1.20]	0.45	[0.24, 0.81]	0.30	[0.16, 0.54]
African American vs. White	0.83	[0.42, 1.51]	1.17	[0.54, 2.35]	1.14	[0.46, 0.88]
Hispanic vs. White	1.31	[0.96, 1.83]	1.16	[0.78, 1.79]	1.71	[1.20, 2.50]
NHPI vs. White	1.99	[1.11, 3.45]	1.17	[0.50, 2.49]	0.96	[0.42, 1.99]
MR vs. White	0.87	[0.50, 1.50]	1.08	[0.55, 2.04]	1.34	[0.74, 2.35]
Other vs. White	1.47	[0.88, 2.41]	1.38	[0.71, 2.58]	1.02	[0.53, 1.90]
Gender						
Male vs. female	1.47	[1.18, 1.85]	1.04	[0.77, 1.39]	1.20	[0.92, 1.54]
Depression						
Depressed vs. not depressed	1.68	[1.30, 2.15]	2.14	[1.54, 2.98]	1.78	[1.34, 2.37]
*AOR *adjusted odds ratio; *NHPI *Native Hawaiian Pacific Islander; *MR *multiracial						

## Discussion

This study examined the psychometric properties of the 5-item SWLS using item response theory from a sample of early adolescents in California. The findings showed excellent item discrimination, difficulty, test information, and conditional reliability across the latent trait, especially among participants who reported below average levels of SWB. Findings also showed concurrent validity as participants who reported high scores on the SWLS, compared to those who reported low scores, were significantly less likely to report lifetime e-cigarette use, cigarette use, and cannabis use. Altogether, these findings suggest that the SWLS may serve as a useful, brief screener of health risk behaviors among early adolescents. A brief measure of SWB may be useful for the design and evaluation of health & well-being programs and interventions that focus on adolescent psychosocial development and mental health.

Past research has supported the reliability, dimensionality, and concurrent and predictive validity of the SWLS with desirable outcome measures, as well as provided scale translations into Arabic, Brazilian-Portuguese, and Chinese, among other languages (Chinni & Hubley, 2014). While most of this research, particularly among adolescents (Jovanović, 2016), has provided psychometric support using CTT, this study advanced the measurement literature on the SWLS by using item response theory. For example, item response models showed that items adequately differentiated between those high on the SWLS compared to those low, and that reliability and test information were most useful among participants between 2 standard deviations below the mean and the mean. Option characteristics curves also showed that most items had separation along the latent trait. There was no evidence suggesting a need to adapt or omit any poor-performing items in future youth SWLS research. These findings indicate that the SWLS may be an accurate measure of well-being among early adolescents, especially for those adolescents who may be struggling with their mental health.

Past research has shown that adolescents who reported low life satisfaction, compared to those high in life satisfaction, were significantly more likely to report delinquency (e.g., truancy, sexual relationships, fighting, leaving home without parental consent, stealing), which was found to mediate the relationship between life satisfaction and substance use behaviors (Mohamad et al., 2018). Other research has found that low life satisfaction scores increased the odds of reporting lifetime tobacco, alcohol, and cannabis use (Lew et al., 2019; O’Donnell et al., 2022). These findings extend past research by showing that participants who reported high scores on the SWLS, compared to those who reported low scores, were significantly less likely to report lifetime e-cigarette use, cigarette use, and cannabis use.

The self-medication hypothesis suggests that individuals are more prone to developing problematic substance use behaviors due to a tendency to cope with negative internal experiences (Hawn et al., 2020). During adolescence, heightened emotional variability and underdeveloped cognitive control systems amplify the risk of maladaptive coping mechanisms like substance use. Low life satisfaction during this stage could increase the propensity for self-medication behaviors as an immediate relief mechanism, highlighting the importance of interventions that foster positive emotions and develop effective coping strategies.

Future research should examine the predictive validity of the 5-item measure of SWLS with other health risk behaviors, such as past 30-day substance use, as well as examine the discriminant validity of the SWLS with other well-being measures (e.g., Seligman’s PERMA model) (Seligman, 2018). Longitudinal studies that use a retrospective/prospective cohort study design may be needed to examine to what extent SWB is a protective factor against tobacco and cannabis use initiation among adolescents throughout their high school education and beyond, especially among youth who are coming of age in the wake of the Coronavirus Disease pandemic of 2019. Future research may also examine measurement invariance across variables of interest (e.g., ethnicity, gender).

### Limitations

Data were collected from adolescents in California and may not generalize to adolescents in other regions of the U.S. Data were collected in 2013, and samples with data collected more recently may be useful to determine if the SWLS psychometric performance continues to be as strong as it was in this study. Nonetheless, the SWLS has been validated with measurement invariance across populations for decades. Data were collected using a self-reported cross-sectional survey of a positive construct. Research has shown that the perceived positive value of a construct may lead to social desirability bias on survey instruments (Longo et al., 2018). Thus, research that includes measures of negative affect in addition to positive affect may be helpful, as well as research designs that control for methodological biases (Donaldson et al., 2021). This study did not collect data on the severity of substance use behaviors or whether use was current. Instead, substance use was measured as lifetime use, which limits the ability to distinguish between experimental, occasional, or frequent use patterns. Future research should include measures that assess the frequency and intensity of substance use to better understand its relationship with life satisfaction.

## Conclusion

This study provided support for the psychometric properties of the 5-item SWLS using item response theory. Findings from the item and test level information advanced measurement precision on the SWLS and showed preliminary associations between the SWLS and tobacco and cannabis use among early adolescents. These findings provide a more nuanced understanding of how the SWLS performs across different levels of life satisfaction among adolescents, informing more targeted and effective well-being interventions.

## Data Availability

Data will be made available upon request.
